# Electrospun poly(3-hydroxybutyrate-co-3-hydroxyvalerate) scaffolds – a step towards ligament repair applications

**DOI:** 10.1080/14686996.2022.2149034

**Published:** 2022-12-19

**Authors:** Thammarit Khamplod, James B. Winterburn, Sarah H. Cartmell

**Affiliations:** a Department of Chemical Engineering, School of Engineering, Faculty of Science and Engineering, The University of Manchester, Manchester, UK; b Henry Royce Institute, The University of Manchester, Manchester, UK; c Department of Materials Science, School of Natural Sciences, Faculty of Science and Engineering, The University of Manchester, Manchester, UK

**Keywords:** Tissue engineering, anterior cruciate ligament, electrospinning, poly(3-hydroxybutyrate-co-3-hydroxyvalerate), fibroblast, cell attachment

## Abstract

The incidence of anterior cruciate ligament (ACL) ruptures is approximately 50 per 100,000 people. ACL rupture repair methods that offer better biomechanics have the potential to reduce long term osteoarthritis. To improve ACL regeneration biomechanically similar, biocompatible and biodegradable tissue scaffolds are required. Poly(3-hydroxybutyrate-co-3-hydroxyvalerate) (PHBV), with high 3-hydroxyvalerate (3HV) content, based scaffold materials have been developed, with the advantages of traditional tissue engineering scaffolds combined with attractive mechanical properties, e.g., elasticity and biodegradability. PHBV with 3HV fractions of 0 to 100 mol% were produced in a controlled manner allowing specific compositions to be targeted, giving control over material properties. In conjunction electrospinning conditions were altered, to manipulate the degree of fibre alignment, with increasing collector rotating speed used to obtain random and aligned PHBV fibres. The PHBV based materials produced were characterised, with mechanical properties, thermal properties and surface morphology being studied. An electrospun PHBV fibre mat with 50 mol% 3HV content shows a significant increase in elasticity compared to those with lower 3HV content and could be fabricated into aligned fibres. Biocompatibility testing with L929 fibroblasts demonstrates good cell viability, with the aligned fibre network promoting fibroblast alignment in the axial fibre direction, desirable for ACL repair applications. Dynamic load testing shows that the 50 mol% 3HV PHBV material produced can withstand cyclic loading with reasonable resilience. Electrospun PHBV can be produced with low batch variability and tailored, application specific properties, giving these biomaterials promise in tissue scaffold applications where aligned fibre networks are desired, such as ACL regeneration.

## GRAPHICAL ABSTRACT

**Figure:**
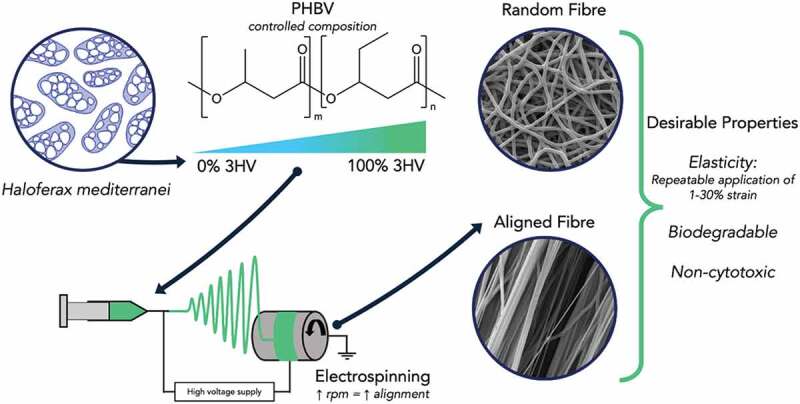

## 1. Introduction

Tissue repair and regeneration is a complex process involving an understanding of cell biology and materials science in order to proliferate neo healthy tissue on suitable materials [1]. The physical properties of a biomaterial play an important role in promoting the tissue regeneration process and these desired properties vary depending on the type of target tissue. Anterior cruciate ligament (ACL) injuries are often encountered as a consequence of general accidents, such as trips and falls, and more commonly in sports, especially in collegiate athletics [2]. ACL reconstruction treatment is available for those who are injured, however, issues such as re-rupturing of the ACL and inefficient movement of the knee are often encountered after surgery [2,3]. Hence, there is a requirement to develop effective biomaterials to support ACL reconstruction for overcoming those problems. Close matching of mechanical properties and mimicking of the ACL structure are key parameters which any potential biomaterials must possess [4,5]. The human ACL consists of bundles of microfibrils, subfibrils and fibrils called fascicles which have diameters in the range of 100–250 µm [6,7]. The mechanical properties of the ACL vary depending on age and gender, typical ACL properties, for the young human group (age between 22–35 years), are an elastic modulus of 128 ± 5 MPa, an ultimate tensile strength of 2160 ± 157 N, a stiffness of 242 ± 38 N/mm and an energy absorbed of 11.6 ± 1.7 N.m [8,9]. The scaffolds currently used for ACL repair supply adequate mechanical properties, but lack of mechanical stability over time and unmatching with native tissue are obstacles [10].

Many attempts have been made to fabricate a variety of suitable materials for ACL scaffolds, exploiting both natural and synthetic materials. Silk-based scaffolds have been widely used in ACL regeneration due to silk’s hierarchical textile structure with similarity to the ACL structure [11]. There are challenges in using silk, including deterioration of silk during the degumming process [12,13]. Synthetic biodegradable polymers, such as polyglycolic acid (PGA), poly-L-lactic acid (PLLA) and poly(lactic acid-co-glycolic acid) (PLGA) have been investigated and found to be promising tissue scaffold materials for ACL repair, however the loss of mechanical strength during biodegradation and rapid degradation rates are significant issues which need to be better understood [11,14,15].

Polyhydroxyalkanoates (PHAs) are a type of biodegradable polyester, biosynthesised by a range of microorganisms such as *Cupriavidus necator* [16,17], *Pseudomonas putida* [18], *Halomonas halophila* [19], *Haloferax mediterranei* [20,21]. The key advantageous properties of PHAs are greater biocompatibility and non-toxicity of degradation products. These properties have led to a range of applications, such as biomedical and food packaging [1,22–24]. There are some examples in which the potential of poly(3-hydroxybutyrate-co-3-hydroxyvalerate) (PHBV) in biomedical applications has been investigated, including drug delivery, bone tissue engineering, wound healing and ligament tissue engineering [1,25–31]. For these applications, PHBV were mostly fabricated by electrospinning, a conventional method used to fabricate materials (especially polymers) into fibre or membrane structures by using electrostatic force to overcome the surface tension of the polymer in solution and induce an elongated jetted fibre from the syringe towards the collector, in order to adapt their structure to allow for specific tissue proliferation and to increase the mechanical strength of the scaffold and handle tissue loading during the regeneration process [32,33]. In ACL engineering, the mechanical properties of film cast PHBV are inadequate, and the film architecture is unable to mimic ACL structure for cell proliferation, failing to meet the basic requirements for an engineered ACL regeneration material [7,34].

In previous work, the mechanical and thermal properties of PHBV were broadly studied and reported [20]. The improved elasticity of PHBV films compared to PHB has been demonstrated, however, to meet the requirements of ACL scaffold material, the fabrication of aligned fibre PHBV and positive in vitro test need to be accomplished. In addition, the mechanical strength and elasticity need to be investigated to provide assurance that PHBV is adequate as a scaffold material for the ACL regeneration process. This work therefore focuses on understanding the relationship between the mechanical and thermal properties of PHBV electrospun fibre mats and the fermentation and electrospinning conditions used. A range of PHBV copolymers with varying 3HV fractions were produced and subsequently electrospun in order to identify the most suitable combination of production conditions (3HV fraction and electrospinning parameters), in terms of mechanical strength, elasticity, surface morphology and biocompatibility for generation of scaffolding materials, feasible for the use in ACL tissue engineering applications.

## 2. Experimental details

The driving rationale behind this work was the exploration of PHBV as a tissue scaffold material for ligament tissue engineering. Experiments were designed to biosynthesise a range of PHBV with varying 3HV content, characterisation of the PHBV produced (3HV content, structure), material fabrication by electrospinning PHBV and the subsequent determination of surface morphology, mechanical (tensile testing and dynamic loading) and thermal properties of the electrospun fibre mats produced. The assessment of these properties performed was used as a basis for selection of the PHBV biomaterial most suitable and able to meet the requirements for ACL repair material. Finally, cell viability testing was carried out on different fibre orientations to observe cell proliferation behaviour and PHBV fibre interaction, to demonstrate the suitability of PHBV as a biomaterial for ACL scaffold applications.

### 2.1 PHBV biosynthesis

PHBV biosynthesis was carried out according to previous research [20]. Briefly, Haloferax mediterranei DSM 1411, obtained from the Leibniz Institute DSMZ-German Collection of Microorganisms and Cell Cultures, was employed to biosynthesise PHBV biopolymers across a wide range of 3HV composition, using minimal synthetic media (MSM) with volatile fatty acids, butyric and valeric acid (Sigma Aldrich, UK), as carbon substrates.

### 2.2 PHBV extraction and purification

To recover the intracellular PHBV, culture medium was centrifuged at 7000 rpm for 10 minutes (Sigma 6–16S, SciQuip, UK) and the supernatant (growth media) discarded. The cell pellet was then re-suspended in 0.1% w/v of sodium dodecyl sulphate (SDS) (Sigma Aldrich, UK) and agitated for 30 minutes for cell lysis. The cells were centrifuged and re-suspended 5 times until a white pellet was obtained and were finally washed with distilled water to remove the SDS. Then, the white pellet was dissolved in chloroform (CHCl_3_) (Sigma Aldrich, UK) at 10 g/L for at least 24 hours. Subsequently, the polymer solution was slowly dropped into stirred cold ethanol using a glass Pasteur pipette in order to precipitate the PHBV. The solid PHBV was separated from the ethanol-chloroform mixture by decantation and solvent evaporation.

### 2.3 Nuclear magnetic resonance (NMR)

To determine the biopolymer structure, the copolymer was characterised by ^1^H and ^13^C NMR, respectively. To prepare the PHBV samples for NMR, 40 mg of PHBV were dissolved in CDCl_3_ (Sigma Aldrich, UK) and were then transferred to NMR tubes (Norell_®_, UK) for analysis with a B500 MHz Avance II+ (Bruker, USA). The D value was calculated as shown in Equation 1, briefly, the peak areas of homo-neighbourhoods of 3HB-3HB and 3HV-3HV are compared with the peak areas of hetero- neighbourhoods of 3HB-3HV and 3HV-3HB in order to give an indication of structure. For a random copolymer the D value is close to 1, for block copolymer D > 1 and for alternating copolymer D < 1 [20,35].

(1) $$D=F_{HB*HB}\times F_{HV*HV}/F_{HB*HV}\times F_{HV*HB}$$

where, F_HB*HB_ is fraction of 3HB adjacent to 3HB monomers, F_HV*HV_ is fraction of 3HV adjacent to 3HV monomers and F_HB*HV_ is fraction of 3HB adjacent to 3HV monomers.

### 2.4 Electrospinning

The electrospinning apparatus was set up with a rotating aluminium collector, automatic syringe pump (Cole Parmer_®_, UK) and power supply (GLASSMAN_®_ high voltage, inc., UK). A 10 mL glass syringe was loaded with 5% w/v PHA solution in chloroform and equipped with stainless-steel metal needle (21 gauge). The metal needle and collector were connected to a positive electrode and ground. The flowrate, supplied voltage and tip-to-collector distance were maintained at 1.0 mL/h, 12kV and 12 cm. After electrospinning the fibre mat was peeled off from the aluminium foil and was kept in desiccator.

### 2.5 Surface property characterisation

Electrospun fibre mat morphology was characterised by scanning electron microscopy (SEM; Tescan Vega 3, UK). The membranes were sputter-coated with gold (Au) under vacuum at 50 mA (Emitech K550×, UK). The SEM was operated at 10 kV at magnification from 500× to 2000 × . Fibre diameter was quantified from image processing of SEM images obtained at 1000× magnification using ImageJ software.

The surface wettability was measured by a surface tension measurement (Kruss 100 Tensiometer, UK) at room temperature. To determine water contact angle, 1.5 µL of deionised water were dropped onto the PHBV fibre mats at four different locations and an average value taken.

### 2.6 Characterisation of thermal properties

Thermal properties of PHBV were characterised using a differential scanning calorimeter DSC (Q100, TA Instruments, UK). At least 2.5 mg of purified PHBV were placed in an aluminium cup and heated to 200°C at 10°C/min, maintained at this temperature for 1 min, then cooled to −20°C at 10°C/min, heated up to 200°C at 10°C/min and cooled to −20°C. Glass transition temperature (T_g_), melting temperature (T_m_) and crystallisation temperature (T_c_) were determined temperature at slope change, temperature mid exothermic peak, temperature mid endothermic peak, respectively.

### 2.7 Wide-angle X-ray diffraction (WAXD)

The WAXD was measured using a Bruker D8 PSD in 2θ range of 5-60° at scanning speed of 1°C/min. The Copper Line Focus X-ray tube with Ni kβ (K_α1_ = 1.540598 Å, K_α2_ = 1.544426 Å, K_α_ ratio 0.5, K_αav_ = 1.541874 Å) was employed as a radiation source. The degree of crystallinity was calculated using Bragg’s law.

### 2.8 Mechanical testing

The electrospun fibre mat was cut into a rectangular shape with dimensions 5 × 25 mm and film thickness measured using a micrometre. The mechanical properties were characterised using a universal testing machine (3344 Testing system, Instron, USA) equipped with 10 N load cell and a tensile rate of 1 mm/min at ambient temperature. Cyclic testing was carried out with an ElectroForce_®_ Planar biaxial testing machine (TA instruments, UK) equipped with 22 N load cell and 3 mm/min in wet condition. Resilience was calculated from the area of loading and unloading stress-strain curves as per the following equation 2.

(2) %Resilience=Area under unloading curve/\breakArea under loading curve×100

### 2.9 Cell culture

Mouse fibroblast L929 were cultured in Dulbecco’s Modified Eagle Medium (DMEM) containing 10% fetal bovine serum (FBS) (Gibco, UK) in the presence of 10 µg/mL antibiotic. The cell line was seeded in 75 cm^2^ cell culture flasks and incubated at 37°C in humidified condition containing 5% CO_2_. Before seeding cells onto samples, cells were trypsinised with 0.05% trypsin in EDTA solution (Gibco, UK) and were then washed with PBS buffer (Thermo fisher, UK) and seeded on to samples in 24-well plate with 2 × 10^4^ cell/well.

### 2.10 Cytotoxicity testing

The electrospun fibre mats were cut into circular dimensions of 10 mm diameter. The fibre mats were washed 3 times with 70% v/v ethanol and were exposed with UV-light for 2 hours in order to sterilise. Before cell seeding, the scaffolds were washed three times with PBS buffer and were immersed in DMEM supplemented with 10% bovine serum (FBS) and 10 µg/mL antibiotic overnight. At days 1, 5 and 7, samples seeded with cells were measured to determine cytotoxicity using the Alamar blue and Hoechst DNA assays. To prepare the Alamar blue solution, 5 mg of Resazurin salt (Sigma, UK) were dissolved in PBS and filter sterilised via a 45 µm Nylon filter. One mL of Alamar blue and DMEM (ratio, 1:9) was added in each well plate and then incubated for 2 hours. Once incubated, the DMEM from each well was transferred to a 96-well plate (in quadruplicate) and growth was measured using a fluorescent microplate reader (Fluostar Optima, UK) at 510 nm of excitation and 590 nm for emission wavelengths. The samples were tested in quadruplicate at each time point.

The Hoechst DNA assay was used to quantify the number of nucleotides in cell cultured on materials. Briefly, the Hoechst working solution was prepared by dissolving Hoechst stock solution (Invitrogen) in TNE (Tris-NACL-EDT) buffer (ratio 1:200). Culture medium was removed from a sample well and transferred to 1.5 mL Eppendorf tubes. 500 µL Nuclease-free water (Sigma, UK) was added to each sample tube, then vortexed and frozen at −80 ºC for 10 minutes. This cycle was repeated three times, following which samples were centrifuged. 50 µL of supernatant liquid were transferred to a 96-well plate (in quadruplicate) to which 50 µL Hoechst working solution were added per well. The plate was read by a fluorescent microplate reader (Fluostar Optima, UK) at 355 nm for excitation and 460 nm for emission wavelengths. The samples were tested in quadruplicate at each time point.

### 2.11 Cell morphology observation

Cell seeded samples at days 1, 5 and 7 were washed three times with PBS buffer, and then fixed with 10% formalin in buffer solution for 20 minutes. Samples were permeabilised with 0.1% Triton X-100 in PBS for 5 minutes, followed by a 3× PBS wash. Samples were then stained with Alexa Fluor_®_ 488 (Thermo Fisher, UK) for 1 hour before washing again with PBS prior to staining with diamidino-2-phenylindole (DAPI) (Thermo Fisher, UK) solution for 5 minutes. Cell morphology was observed by confocal microscope (Leica TCS SP8 MP, UK).

### 2.12 Statistical analysis

Data are reported as mean ± standard deviation and were analysed by a one-way ANOVA in different groups of experimental conditions (n = 4). Statistical significance is accepted at 0.05 or 5% level.

## 3. Results and discussion

This work consists of three major parts: (1) PHBV biosynthesis, (2) PHBV electrospinning and determination of mechanical and thermal properties and (3) biocompatibility and cell morphology observation. The relationship between PHBV properties and cell viability on the PHBV biomaterials produced is explored. Based on this the most suitable combination of fabrication conditions, 3HV composition and electrospinning parameters, were selected in order to meet the requirements for ACL scaffold applications.

### 3.1 PHBV biosynthesis and composition

Controllable (3HV fraction) and repeatable PHBV production was accomplished by manipulating the butyric acid and valeric acid supplied to the fermentation via fed-batch strategy which is explained in previous work [20,21]. In this work, shaker flasks of 1 L working volume were used, giving copolymer production in range of 2.0–2.2 g/L and a homopolymer production of 1.4 and 1.6 g/L for PHB and PHV, respectively, see Table 1. The fraction of 3HV in the PHBV copolymer is generally directly proportional to valeric acid addition, however, the actual 3HV content (by mole) of the copolymers produced is somewhat lower than might be expected based on the mole fraction of valeric acid supplied. This is because of limitations of shake flask production (vs. the better controlled bioreactor environment) and the fact the mixed substrate feeding can promote more C5 substrate entering the tricarboxylic acid cycle (TCA) in order to produce chemical energy for cell growth and reproduction instead of PHA production, resulting in a decrease of 3HV fraction in the copolymer [36].

**Table 1.** 3HV content, productivity and thermal properties of PHBV for fed-batch culture with various mixed C4:0/C5:0 substrates.

Experiment	Actual 3HV		Productivity	Molecular weight	T_g_	T_c_	T_m_	Enthalpy (∆H_f_ (T_m_))
	(% by mol)	D Value	(mg.l^−1^.h^−1^)	(10^6^g/mol)	(°C)	(°C)	(°C)	(J/g)
0% 3HV	100	n.d.	0.02	4.70	n.d.	n.d.	173.9	84.8
25% 3HV	20	0.94	0.04	3.61	−3.0	69.8	135.2, 167.2	57.5
50% 3HV	43	1.97	0.04	3.75	−6.3	n.d.	n.d	n.d
75% 3HV	71	1.57	0.04	3.80	−10.6	n.d.	n.d	n.d
100%3HV	≈100	n.d.	0.03	2.27	−18.6	42.3	107.2	54.1

**Figure 1.**
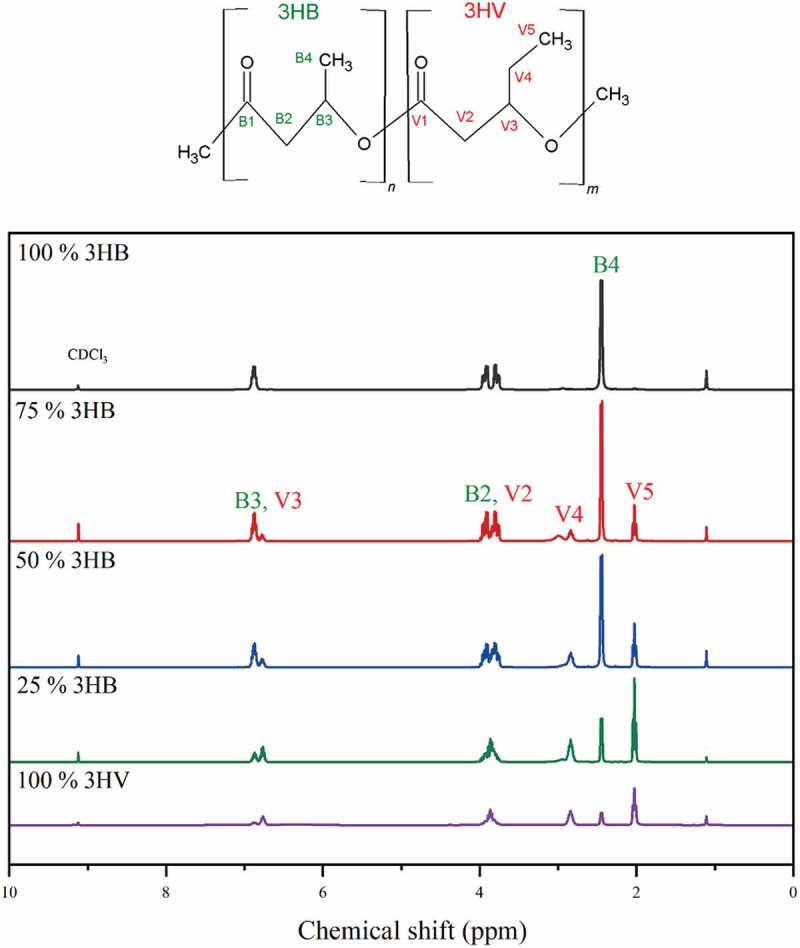
^1^H chemical shifts for PHB, PHV and PHBV at 500 Hz.

^1^H NMR results are shown in Figure 1, the chemical shifts (δ) of central hydrogen atom represent the different positions of hydrogen atom in PHBV sub-unit. The hydrogen atoms, B1-B4 and V1-V5, represent the 3HB and 3HV sub-units of PHBV, respectively. For the 3HV units in the copolymer, the proton resonance of CH_3_-, and CH_2_- groups are determined as chemical shifts of 0.82 and 1.56, respectively. This confirms that there is 3HV in the copolymer formed, PHBV. To determine the structure of PHBV produced ^13^C NMR was conducted, as in previous work [20], which confirms that as expected a random PHBV copolymer is produced from a C4:0/C5:0 co-feeding strategy when PHBV is synthesised by Haloferax mediterranei and From Table 1, D values of copolymer production which is closing to 1 indicates that the copolymers from 25% to 75% valeric acid co-fed are random copolymer [20].

### 3.2 PHBV film morphology

The PHB, PHBV and PHV film morphologies are shown in Figure 3. A ‘grain-like structure’ surface is observed for pure PHB, 3HB rich PHBV and pure PHV films [37]. The grain like structure is formed because of the high degree of crystallinity in high purity polymers, as confirmed by the high heat of fusion values measured, see Table 1. A smoother surface is observed in PHBV copolymers of 50% and 70% 3HV because of their amorphousness. The amorphous nature of 3HV rich copolymers is due to the steric effect of the longer side chain of 3HV, with both 3HB and 3HV units try to rearrange in the same crystal lamellae resulting in higher disorder in the copolymer structure [38].

**Figure 2.**
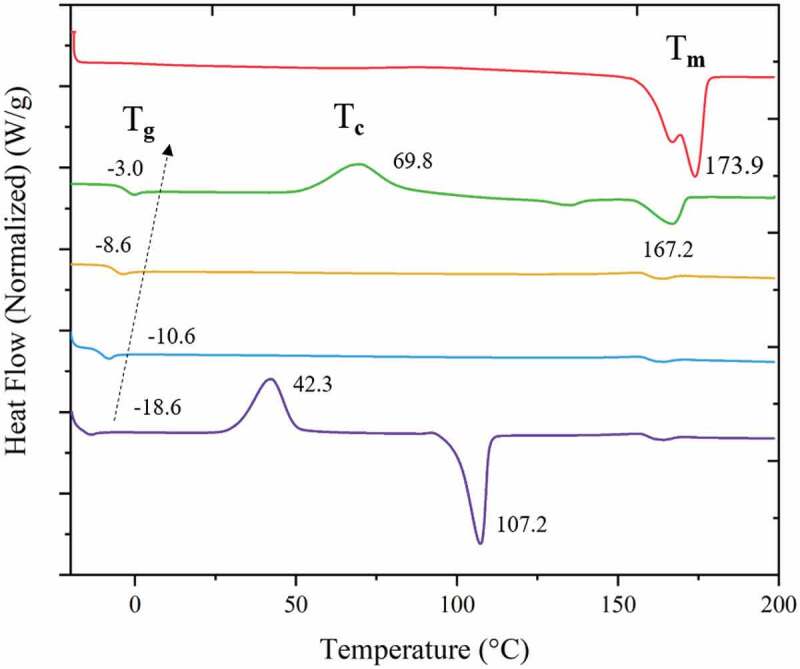
Second-heating scan thermogram of −100% 3HB (red), 75% 3HB (green), 50% 3HB (yellow), 25% 3HB (blue) and 100% 3HV (purple) - PHBV.

**Figure 3.**
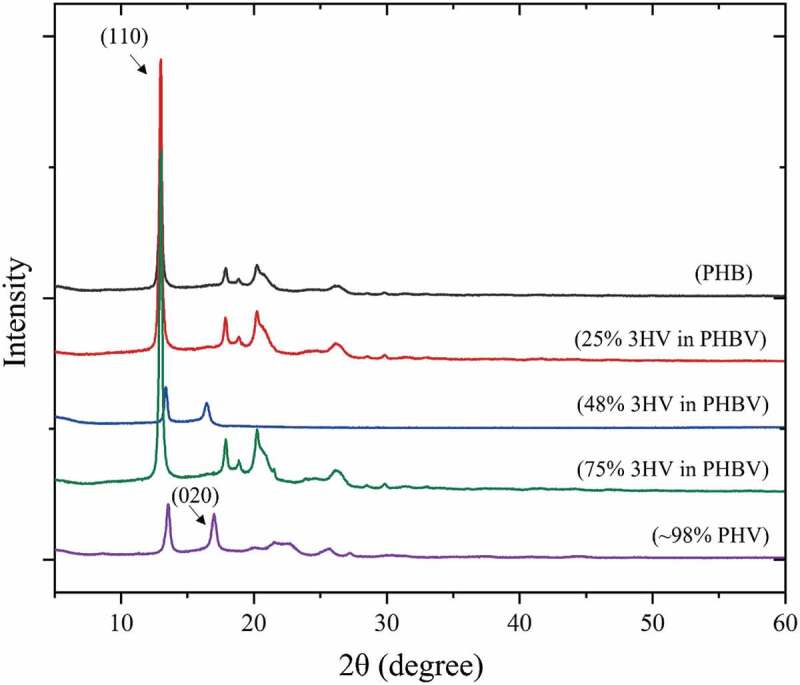
Wide-angle X-ray diffraction patterns for 100% 3HB (red), 75% 3HB (green), 50% 3HB (yellow), 25% 3HB (blue) and 100% 3HV (purple) - PHBV.

### 3.3 PHBV thermal properties and crystallinity

Thermal properties of the PHBV copolymers produced are reported in term of glass transition temperature (T_g_) and melting temperature (T_m_), as shown in Figure 2 and Table 1. To assess the degree of crystallinity, the heat of fusion of the polymer is compared to the heat of fusion of completely crystalline polymer (∆H^0^_f_(T^0^_m_)) which is 146 J.g^−1^ [39]. Therefore, for all PHBV compositions studied the copolymers are amorphous. However, at 25 mol% 3HV PHBV exhibits a double melting temperature (T_m_) and a crystallisation temperature (T_c_) at 135.3/167.2°C and 69.8°C, respectively. This means that there existed a major single crystal of PHB in the copolymer, which is confirmed by the T_c_ corresponding to the cold crystallisation temperature (Figure 2) of pure PHB (≈70 °C) [38]. For this reason, the film morphology of 25 mol% 3HV PHBV also forms a grain-like structure [40]. The degradation temperature (T_d_) of pure PHB is reported in the literature to be approximately 220 °C [41]. The difference between T_m_ and T_d_ is defined as the temperature processing window of a polymer, which can be improved by decreasing T_m_. Incorporation of additional 3HV units in the PHBV copolymer could decrease T_g_ and T_m_ which in turn widens their temperature processing window [42].

The WAXD pattern in Figure 3 shows a peak at 2θ = 13° which represents the (110) diffraction of 3HB homopolymer-type lattice with a further peak at 2θ = 18° which represents the (020) of the 3HV homopolymer type-lattice. The PHBV copolymer shows both characteristic peaks of PHB and PHV which means that the two single crystals exist in the PHBV copolymer [43]. At 50% 3HV in PHBV the pattern shows the less crystalline peaks than for the others. This could confirm the lower T_m_ peak of 50% 3HV in PHBV in Figure 2.

### 3.4 Electrospinning conditions, morphology, and thermal properties

**Figure 4.**
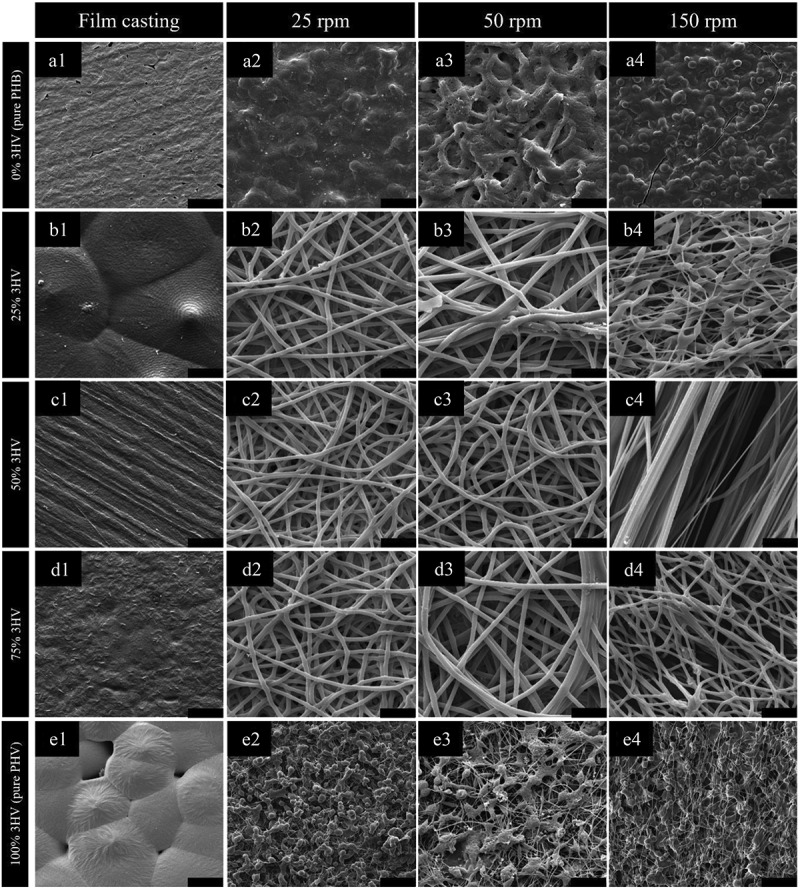
SEM micrographs of PHBV with 0 to 100% (A1 to A4 and E1 to E4) 3HV compositions (by mole) and 25 to 150 rpm of rotating speeds (B1 to B4, C1 to C4 and D1 to D4) (scale bar = 50 µm).

PHBV copolymers with 3HV contents of 0, 25, 50, 75 and 100 mol%, were electrospun under controlled conditions with the collector rotational velocity varied from 25 to 50 and 150 rpm. Collector rotational velocity was varied in order to give understanding of the formation of PHBV fibres. To observe the fibres formed and their orientation and degree of alignment, the fibres were imaged, and the resulting 1000× SEM micrographs are shown in Figure 4. From the quantitative analysis of fibre orientation (Figure S2 in supporting information) for the electrospun fibre mats produced at rotational speeds of 25 and 50 rpm, the fibres have a random pattern and low to non-existent amount of alignment compared to the fibre mats obtained at 150 rpm, across all 3HV contents.

When pure PHB or PHV were electrospun, ‘a webbing’ and ‘a bead-like structure’ were formed, Figures 4A and 4E. The irregular fibre structure is formed due to the initial low viscosity of the polymer solution. Although biosynthesised PHB has a higher molecular weight compared to PHBV, see Table 1, pure PHB/PHV have very high crystallinity which leads to requiring more time for de-crystallisation and consequently partial dissolution in the initial PHB/PHV solution for electrospinning [44,45]. This consequently affected the electrospinning process, caused by incomplete solvent evaporation with the crystalline regions also formed in the elongated jet stream resulting to ‘webbing’ and ‘bead-like structure’ in the PHB/PHV electrospun fibre mat [46].

The morphology of electrospun PHBV fibres with different 3HV fractions is demonstrated in Figure 4 (B1-D4), with the smoothest fibres obtained at 50% and 75% 3HV. The fibre diameter of random (25 rpm and 50 rpm) and aligned fibres (150 rpm) are 2.420, 2.910 and 2.296 µm, respectively. The fibre diameter of random copolymer is significantly higher than in aligned fibre copolymer because at higher rotating speed the polymer jet elongated more than at lower rotating speeds, resulting in a reduction in the fibre diameter [47]. The variation in PHBV fibre diameter of 50% and 75% 3HV is lower than for other compositions, due to the presence of amorphous regions in the bulk polymer resulting in a homogeneous initial polymer solution which promotes a continuous polymer jet, with fewer crystalline regions to hinder polymer jet stretching [48].

Considering the aligned fibres produced at 150 rpm the 50 mol% 3HV electrospun fibre mat shows the highest degree of alignment compared to other compositions. It can be seen that the increasing amorphous region in 50% 3HV copolymer promotes improved alignment in the electrospun fibre mat. In contrast, the stiffer crystalline regions act to retard fibre straightening, which occurred in the 3HB rich copolymer with high crystallinity regions [48]. From the interaction of crystal grains in the copolymer and interruption of tensile force at a rotating speed of 150 rpm, the bead-like structure is formed in 25% 3HV electrospun fibres, shown in Figures 4B–4. A similar trait is also observed in 3HV rich copolymer.

### 3.5 Surface wettability

**Figure 5.**
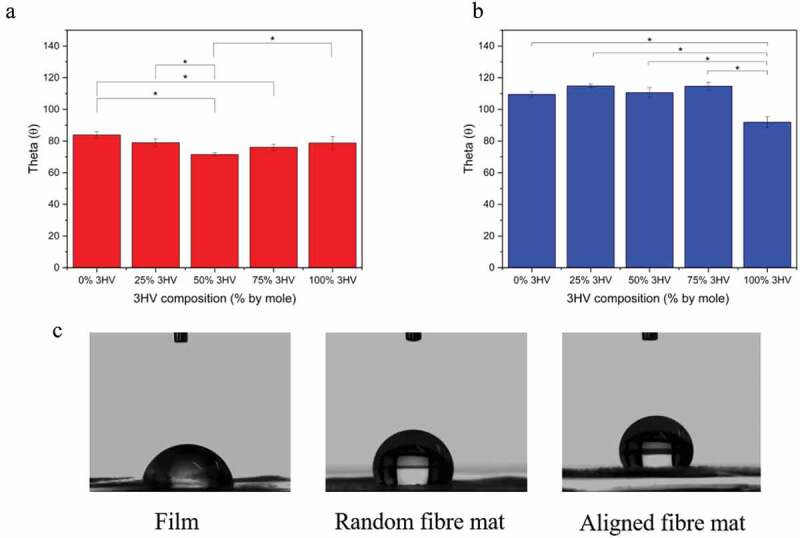
PHBV water contact angle for 0%, 25%, 50% and 75%, 100% 3HV (a) films, (b) electrospun fibres. (n = 4 per condition, *p < 0.05), (c) water droplet images for 50% 3HV PHBV film, random fibre mat and aligned fibre mat.

Surface hydrophilicity of PHBV films and electrospun fibres was characterised by water contact angle measurement. The higher the contact angle the greater the hydrophobicity of the surface. As seen in Figure 5, in general PHBV electrospun fibre mats have higher contact angles, and hence lower hydrophilicity, than PHBV films, due to the smoother surface of films compared to the rougher fibre mat surface. When considering in each type of fabrication, the PHB and PHV cast films have the highest contact angle, 81.4 ± 3.6 θ and 78.7 ± 3.6 θ, respectively. Additional 3HV content reduces the contact angle to 71.8 ± 1.3 θ and 76.0 ± 1.7 θ in 50% 3HV and 75% 3HV copolymers, respectively. In electrospun fibre mats, the water contact angles are higher for pure PHB and PHV, 109.5 ± 1.75 θ and 92.27 ± 4.20 θ, compared to 25% 3HV and 75% 3HV, contact angles of 114.28 ± 5.05 θ and 114.63 ± 2.46 θ, respectively. However, the 50% 3HV electrospun fibre mat has the lowest contact angle of all materials tested, 110.58 ± 3.06 θ. The most influential parameter on surface wettability is submicron surface roughness, which, in turn, is determined by degree of crystallinity in the case of polymer films [49,50]. But in the case of electrospun fibre mats, the nature of the fibril-like structure gives all electrospun samples a similar surface morphology, with the slightly different contact angles a result of different fibre diameters. The larger diameter fibres, as obtained at 50 rpm rotating speed, results in an increase in contact area between water and the material surface, consequently increasing hydrophilicity [27,51].

### 3.6 Mechanical testing

**Figure 6.**
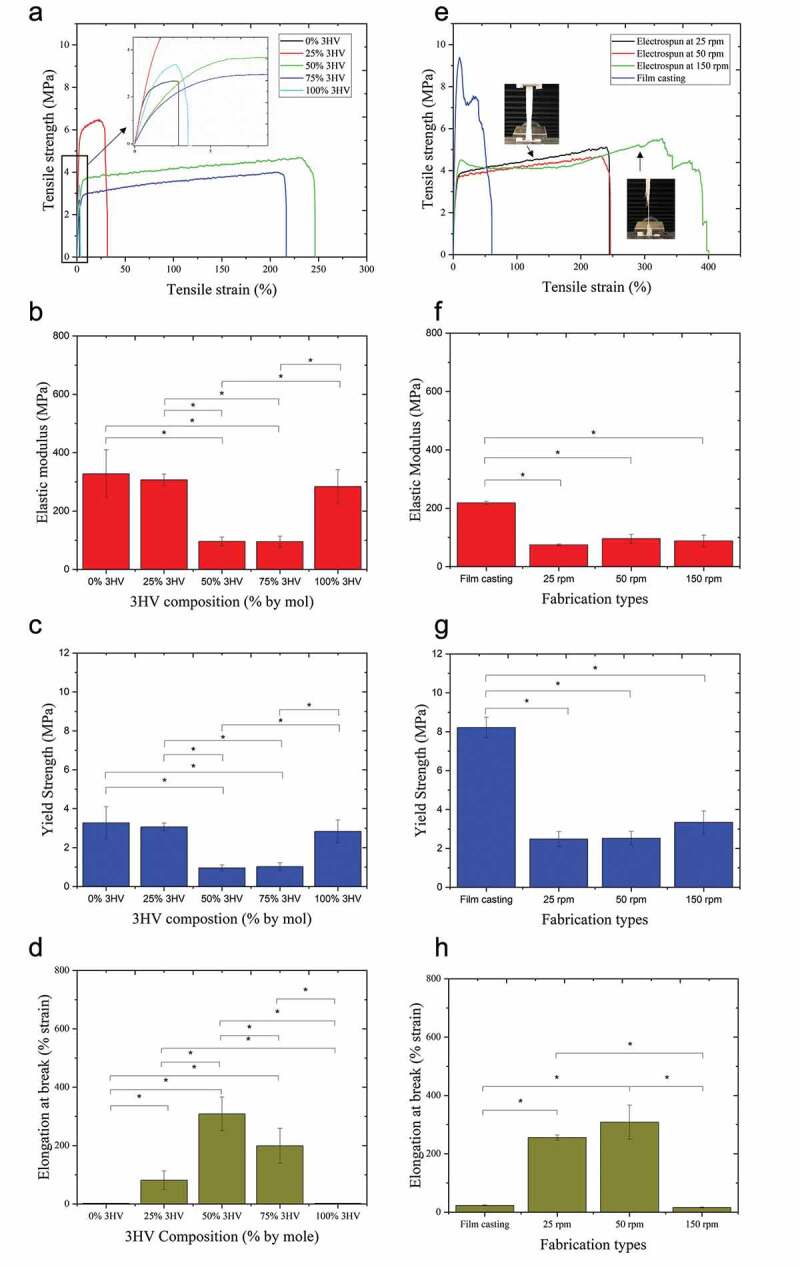
Mechanical properties of PHBV films and electrospun fibres with varying 3HV composition including tensile strength (a and e), elastic modulus (b and f), yield strength (c and g) and elongation at break (d and h). a-d varying 3HV composition, e-h changing fabrication conditions and breaking characteristic between random and aligned fibres (n = 4 per condition, *p < 0.05).

Mechanical properties of electrospun fibres were determined by tensile testing and are reported here as elastic modulus, ultimate tensile strength, and elongation at break, shown in Table 2 and Figure 6. The PHBV stress-strain curve consists of three key stages, characteristic of mechanical testing under tensile loading, which are the toe region, elastic deformation, and plastic deformation regions [52]. The duration of each section varies depending on the mechanical characteristics of the material; brittle, ductile or elastomeric, and so on [53]. Likewise, PHBV electrospun fibre mats also show differences in mechanical characteristic with changing 3HV composition and fibre alignment patterns.

**Table 2.** Electrospun fibre parameters, diameter, water contact angle and mechanical properties (n = 4 per condition, *p < 0.05).

	Fibre diameter	Contact angle	Elastic modulus	Yield strength	Elongation at break	T_m_	Enthalpy (∆H_f_(T_m_)
Rotating speed	(µm)	(θ)	(MPa)	(MPa)	(% strain)	(°C)	(J/g)
25% 3HV							
25 rpm	3.05	114.3	53.3	1.6	223.8	163.2, 184.4	3.6, 0.9
50 rpm	3.60	102.2	306.5	3.6	81.5	162.2, 187.4	6.4. 1.8
150 rpm	1.46 (fibre) 5.02 (bead)	115.8	334.0	3.8	110.6	162.6, 186.3	6.4, 1.7
50% 3HV							
25 rpm	2.42	112.1	44.7	1.7	255.4	n.d.	0.9
50 rpm	2.91	110.6	76.7	1.9	308.4	n.d.	3.8
150 rpm	2.30	114.7	88.2	2.2	15.9	n.d.	1.4
75% 3HV							
25 rpm	2.82	108.8	131.2	1.2	120.9	n.d.	n.d.
50 rpm	3.38	114.6	95.3	1.8	199.0	n.d.	n.d.
150 rpm	2.14	120.5	102.5	0.8	24.1	n.d.	n.d.

The 3HV rich PHBV copolymer, especially at 50% and 75% 3HV, shows the transition from brittle to more elastic behaviour compared to pure PHB, PHV and 25% 3HV PHBV, see Figure 6a. The decrease in elastic modulus represents the improved elasticity of electrospun fibre mat compared to PHBV films which were studied previously [20]. Especially in the case of 50% 3HV PHBV, the elastic modulus of electrospun fibre mat is reduced by a factor of more than two (Figure 6b). This is because the decreased degree of crystallinity in 3HV rich PHBV copolymer at 50 and 75% by mole, confirmed by enthalpy of fusion (∆H_f_(T_m_), as shown in Table 2.

The elongation at break is also undoubtedly improved by electrospinning (Figure 6d). Looking at electrospun samples, the fibre network is arranged randomly or aligned depending on collector rotating speed. The interconnected fibre networks allow the tensile load applied to pass through the interconnecting fibre network and then the force can be suddenly removed by the void or space between fibre network, resulting in the enhancement of elasticity and elongation at break [54]. In Figure 6e, an increasing rotating velocity shows a longer plateau region, which takes place after linear elastic deformation, especially for 150 rpm electrospun fibre mat. The aligned fibres show fibril bundle breaking behaviour under tensile loading which clearly observed as the largest plateau stage, ‘plastic region’. In this stage, the fibril is extended, after which the crystal alignment occurs along the direction of fibre extension which results in increasing tensile force until the extended fibril break [55].

Focusing on 50% 3HV electrospun fibre mat with rotating speeds 25 to 150 rpm, there is no significant difference in the value of elastic modulus and yield strength (Figure 6f,g). In contrast, there is a significant difference in elongation at break between aligned and random fibres (Figure 6h). The aligned fibre shows the lowest elongation at break, 15.9 ± 1.7% (mm/mm) compared to random fibre at 25 and 50 rpm, 255.4 ± 9.3% and 308.4 ± 57.8% (mm/mm), respectively. This is because the characteristic rupture mechanism of aligned fibres involves tearing of fibre bundles aligned within the material, which differs from the case of random fibre networks due to the manner in which the tensile load is distributed in the material sample as seen in Figure 6e.

The different mechanical characteristic in different types of PHBV film, random fibre and aligned fibre mats can be explained by molecular orientation. High yield strength and low elongation at break in PHBV cast films occur because in during film casting process the PHBV chain has more time to allow for crystallisation than in the electrospinning process, which is characterised by rapid solvent evaporation. Among PHBV electrospun fibre mats with different rotating speeds, there is not a significant difference in elastic modulus and yield strength. This is because the degree of crystallisation of those electrospun fibre mats are not obviously different, as seen in Table 2. However, the topography of fibre plays extremely important role in determining mechanical characteristics. In the aligned 50% 3HV PHBV electrospun fibre mat, the stress-strain curve (Figure 6e) of this fibre shows super-wide ‘strain-hardening’ in the plastic deformation region (the curve after linear elastic deformation) similar to tendon and ligament deconstruction [56]. Therefore, 50% 3HV PHBV electrospun fibre mat were selected for in vitro study of cell proliferation, see following section.

**Figure 7.**
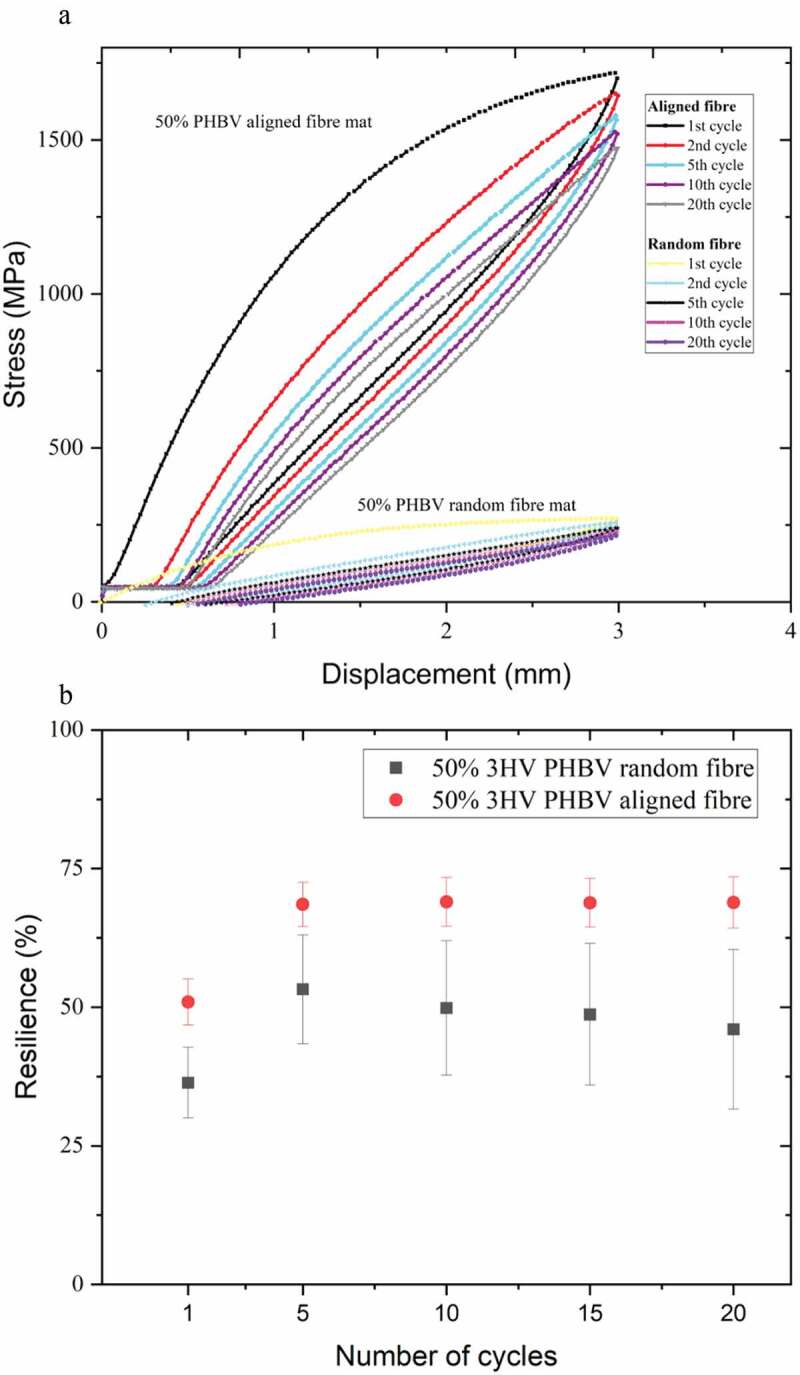
Hysteresis loops (a) and resilience (b) plots of 50% 3HV PHBV aligned fibre and random fibre mats, 1st to 20th cycles under tensile loading/unloading.

Another desirable mechanical property of scaffolds for ligament regeneration is an ability of recovery or energy dissipation upon unloading, that is, resilience [57]. Therefore, cyclic tensile loading-unloading testing was conducted on the electrospun scaffolds to give an indication of material behaviour during long-term implantation under dynamic load conditions. Stress-strain curve cycles and resilience were determined and are shown in Figure 7.

A total of 20 cyclic stress-strain curves of 50% 3HV PHBV random and aligned fibre mats are shown in Figure 7a. At first, cyclic tensile testing shows an irreversible deformation between loading and unloading because of strain-induced crystallisation which can disrupt strain recovery during unloading [58]. Upon the second loading and unloading lesser dissipated energy or higher resilience were observed, this trend continues in subsequent cycles as shown in Figure 7b [59]. The aligned electrospun mat shows higher resilience than does the random electrospun mat, likely due to the higher extensibility and smaller fibre diameter of the aligned fibre mat compared to the random networked fibre mat which has increased crystallinity and thicker fibre [60]. The random fibre mat shows a higher variation of hysteresis, as seen in the larger error bars in Figure 7b. This might be because of an anisotropic property of the random fibre network resulting in less uniform load distribution compared to the aligned fibre mat.

### 3.7 Biocompatibility and cell morphology observation

**Figure 8.**
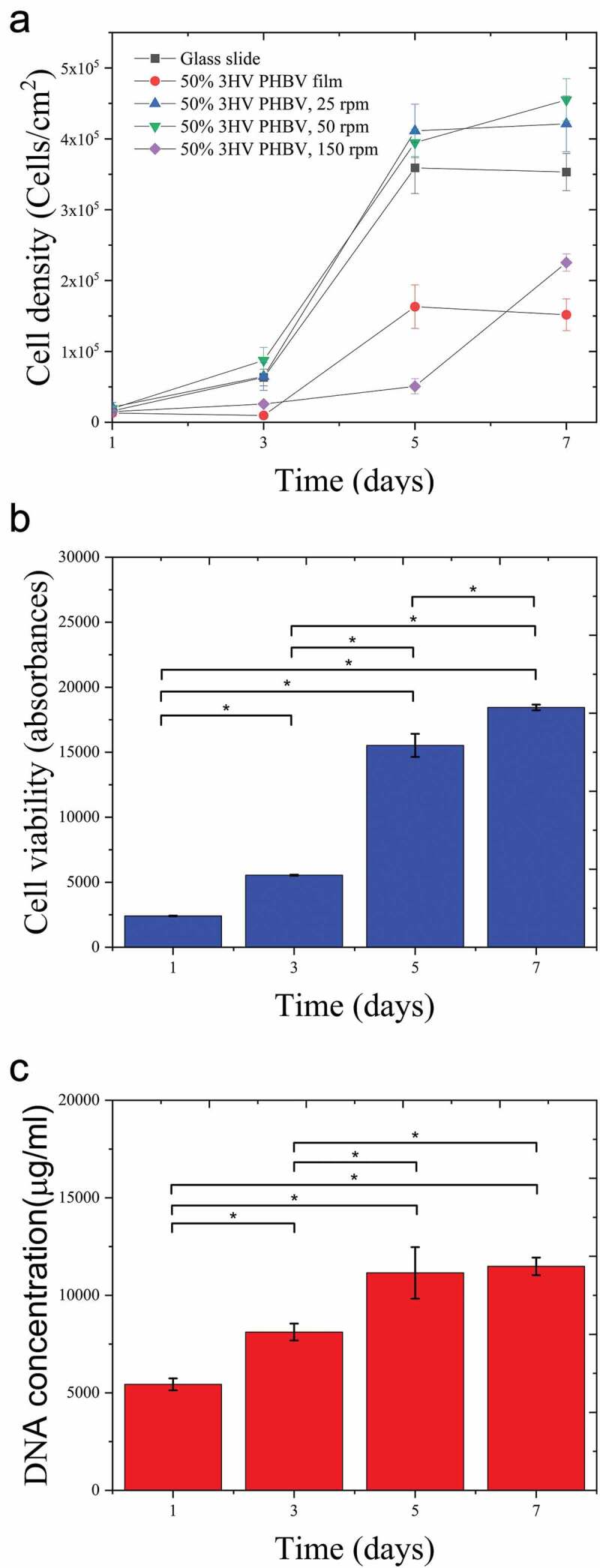
Cell density of L929 fibroblast on materials (Figure 5A) which are glass slide, 50% 3HV PHBV film, 50% 3HV PHBV electrospun fibre mat at 25 rpm, 50% 3HV PHBV electrospun fibre mat at 50 rpm and 50% 3HV PHBV electrospun fibre mat at 150 rpm and quantification of cell viability and DNA using Alamar Blue (Figure 5B) and PicoGreen assays (Figure 5C) for PHBV electrospun fibre mat, culture over 7 days period (n=4 per condition, *p<0.05).

To assess the cell cytotoxicity of PHBV electrospun fibre mats, the proliferation of L929 fibroblast was analysed by Alamar blue and Hoechst DNA assays. Figure 8 shows cell viability and DNA concentration of L929 proliferation on 50% 3HV PHBV electrospun fibre mat at 50 rpm over 7 days. It can be seen that the fibroblast grew on the PHBV electrospun fibre mat, confirming the non-cytotoxicity of PHBV fabricated and biosynthesised by electrospinning and Haloferax mediterranei, respectively.

**Figure 9.**
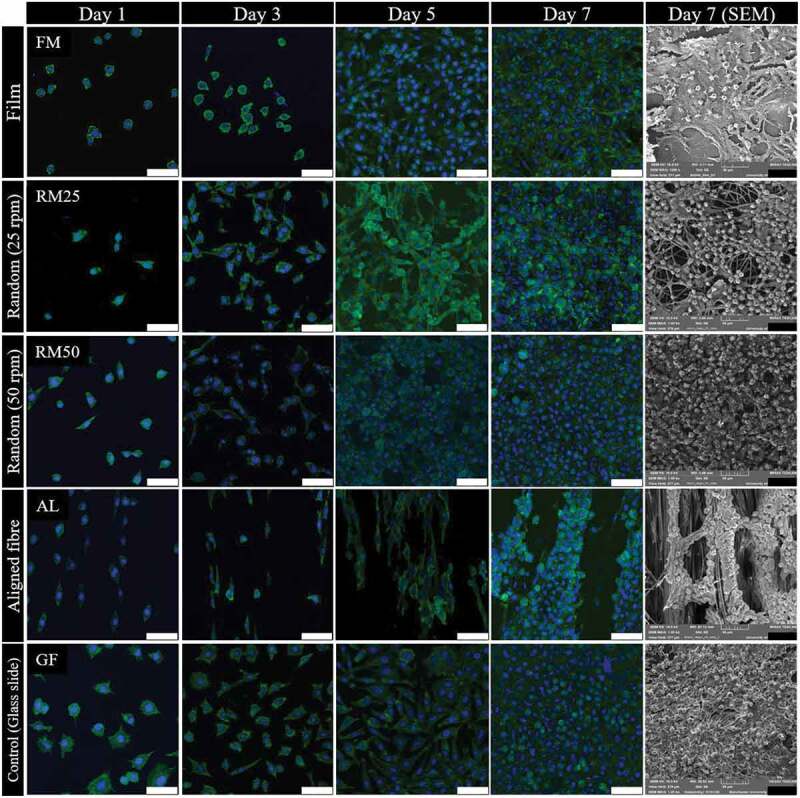
L929 fibroblast strained with phalloidin (green) and DAPI (blue) at 1, 3, 5 and 7 days of culture and L929 fibroblast SEM micrograph at 7 days culture on 50% 3HV in PHBV film (FM), random fibre at 25 rpm (RM25), random fibre at 50 rpm (RM50) and aligned fibre at 150 rpm (AL) in different fabrication conditions (scale bar = 50 µm).

Figure 8a shows the growth curve, cell density against culture time, with a similar growth pattern seen in between both tests, especially the rapid growth from day 3 to 5. In contrast a plateau in cell density exists between days 5 and 7. During days 3 to 5, exponential growth occurred, the changing from rounded shape to spindle-like-shape is observed from day 1 to 3 as seen in Figure 9, then, cells were reproduced and colonised along with the fibre arrangement. During day 5 to 7, cells were not dominantly reproductive, however, cytoplasm expansion is observed instead.

Cell morphologies on different PHBV surfaces are shown in Figure 9. The fibroblast proliferation on 2D materials, glass slide (Figure 9 GF) and PHBV film (Figure 9 FM), are compared to semi 3D materials; electrospun fibre mat produced at 25, 50 and 150 rpm. Comparing between the glass slide and PHBV film, cell proliferation was faster on the slide than on the PHBV film. This is because of hydrophilic surface of the glass slide which favoured cell proliferation [61]. The fibroblast could grow extremely well and spread on fibres both random (at 25 and 50 rpm) and aligned (at 150 rpm), however an obvious difference in cell morphology (Figure 7 RM25, RM50 and AL from 1 to day 5) is found between the random and aligned fibres. For aligned fibres, elongated fibroblasts are observed, orientated in the same direction as the fibre alignment. In addition, the cell growth characteristic is also different on aligned electrospun fibre mat (cell orientation distribution patterns are shown in supporting information, Figure S3).

Figure 8a shows L929 cultured on aligned fibre showed exponential growth occurring later, days 5 to 7, than on the other random fibre samples. This is because of the larger pore size and fewer interconnections in aligned fibre compared to random fibre. Naturally, fibroblast can easily proliferate on a planar axis (x-y planar), therefore, the more interconnected x-y planar network in random fibre could further promote cell proliferation [62]. In contrast, on aligned fibres, more cell infiltration to the bottom of the well-plate compared to random fibre means fewer cells attached to the fibre. Therefore, the cell proliferation in aligned fibre took a longer time to reach the exponential phase.

The 3D images of L929 proliferation on different PHBV topographies (Figure S1 in the supporting information) demonstrate the 3D scan of nucleus (blue) and cytoplasm (green) along with membrane thickness. Using fluorescence imaging, the visual differences in cell proliferation on a glass slide, PHBV film and PHBV electrospun fibre mat were determined. On a glass slide and PHBV film the fibroblast proliferated densely and compactly, however, in electrospun fibre mat random fibre (Figure S1C and S1D in supporting document), cells grew and spread covering the fibre network and inside the pore structure. In the aligned electrospun fibre mat (Figure S1E in supporting document), cells mainly proliferated along the direction of fibre alignment. It could be implied that the aligned PHBV electrospun fibre mat can promote cell infiltration which can physically mimic the extracellular matrix [63,64].

### 3.8 Suitability of PHBV for ACL repair

It is well known that various biodegradable polyesters have been demonstrated as ACL repair materials, for example, polyglycolic acid (PGA), poly-L-lactic acid (PLLA) and poly(lactic acid-co-glycolic acid) (PLGA) [11,14,15]. However, it is not clear which is the most suitable material for this kind of tissue engineering [65]. PHAs have been rarely studied for ligament repair engineering because of limiting mechanical properties, brittleness, and weakness, which are not close enough to the ACL’s mechanical properties [66]. As mentioned before, the ACL mechanical properties are very complex. Therefore, the possibility of using PHBV towards ACL scaffold material is investigated in this work.

From this study, more than 50% 3HV by mole PHBV, aligned electrospun mat shows lower elastic modulus (88.2 to 102 MPa) and higher elongation at break (15 to 24% strain) which means that the super elasticity is provided. Hysteresis testing shows that the resilience is nearly 75% after the first cycle tensile testing and this tends to increase with cycle loading. Nevertheless, the ultimate tensile strength and resilience of the electrospun fibre mat are not yet entirely comparable with the properties of ACL, because the PHBV was fabricated into 2D materials which are obviously different from the 3D structure of ACL tissue, as such there is a need to explore three dimensional PHBV aligned fibre structures, based on the potential of the 2D materials elucidated here. The hierarchical structure of ACL needs to be mimicked by creating complex structures of aligned electrospun mat by, for example, climbing, knitting or braiding [7,67,68].

## 4. Conclusion

A thorough investigation of the variation of PHBV mechanical properties with changing 3HV fraction and electrospinning conditions has led to the identification of an electrospun PHBV aligned fibre mat with 50 mol% 3HV content as a promising candidate scaffold material for ACL repair. Random PHBV copolymers were successfully biosynthesised with a wide range of 3HV compositions and fabricated using an electrospinning technique to produce fibre mat scaffolds. The elastic modulus, ultimate tensile strength, elongation at break and hydrophilicity of the electrospun fibre mats were studied, in order to find a candidate PHBV material which can match the mechanical properties of ACL tissue. A 50% 3HV PHBV electrospun fibre mat exhibits improved mechanical behaviour compared to brittle PHB and PHV polymers, being tougher and more elastic, with aligned fibre mats produced from PHBV having improved resilience compared to random fibre mats. These properties demonstrate the potential of PHBV as a scaffold material and the results obtained move towards the behaviour of natural ACL under dynamic mechanical loading. Biocompatibility tests demonstrate the non-cytotoxicity of PHBV electrospun fibre mat to L929 fibroblasts and the aligned fibre networks promote L929 fibroblast alignment in the fibre direction, which is desirable for ACL repair applications. However, there is a need for fabrication of more complex 3D structures, as a way to achieve better tensile strength of scaffold. The electrospun PHBV scaffold can be produced with low batch variability and properties tailored to suit the specific ACL application, giving the biomaterials promise in ACL regeneration applications where aligned fibre networks are desired.

## Supplementary Material

Supplemental Material
