# Supplementary material for: Electrospun poly(3-hydroxybutyrate-co-3-hydroxyvalerate) scaffolds – a step towards ligament repair applications
Source: Sci Technol Adv Mater. 2022 Dec 19;23(1):895–910. doi: 10.1080/14686996.2022.2149034 (PMC9769142; doi:10.1080/14686996.2022.2149034)

**Supporting Information**

# **Electrospun Poly(3-hydroxybutyrate-co-3-hydroxyvalerate) Scaffolds – A Step Towards Ligament Repair Applications**

Thammarit Khamplod^1^, James Winterburn^1^, Sarah Cartmel,^2,3*^

^1^Department of Chemical Engineering, School of Engineering, Faculty of Science and Engineering, The University of Manchester, Manchester, M13 9PL, UK

^2^Department of Material Science, School of Natural Sciences, Faculty of Science and Engineering The University of Manchester, Manchester, M13 9PL, UK

^3^Henry Royce Institute, The University of Manchester, Manchester, M13 9PL, UK

* Sarah.cartmell@manchester.ac.uk

**Figure S1** Three-dimensional images of L929 fibroblasts stained with phalloidin (green) and DAPI (blue) on A) glass slide, B) PHBV film, and PHBV electrospun at C) 25, D) 50 and E) 150 rpm, all 50% 3HV.

**
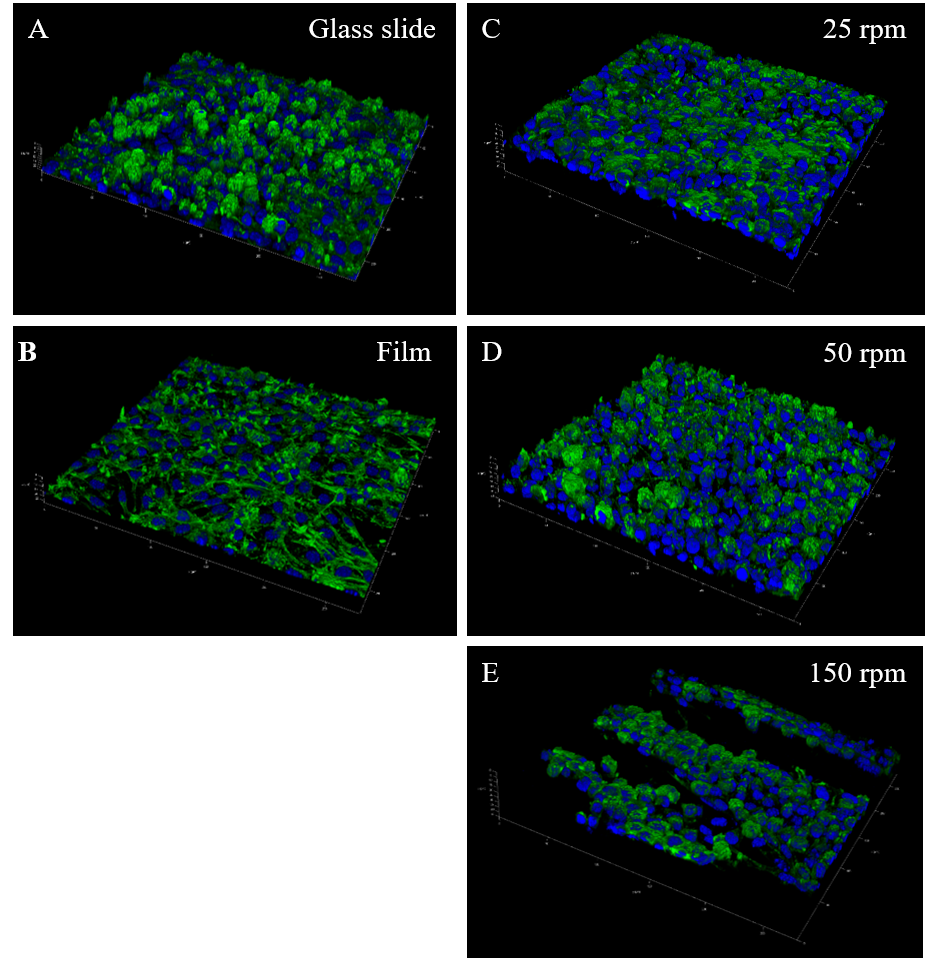
**

**Figure S2** Fibre orientation distribution curves (x-distribution of orientation and y-orientation in degree) for electrospun PHBV with varying 3HV content: A1 to A3, 25 mol% 3HV spun at 25, 50, and 150 rpm, B1 to B3 50 mol% 3HV, 25, 50, and 150 rpm and C1 to C3 75 mol% 3HV, 25, 50, and 150 rpm. Images were analysed with ImageJ. using OrientationJ. The multiple peaks of orientation degree represent less aligned fibre orientations, whilst sharper individual peaks represent alignment of fibre orientation.


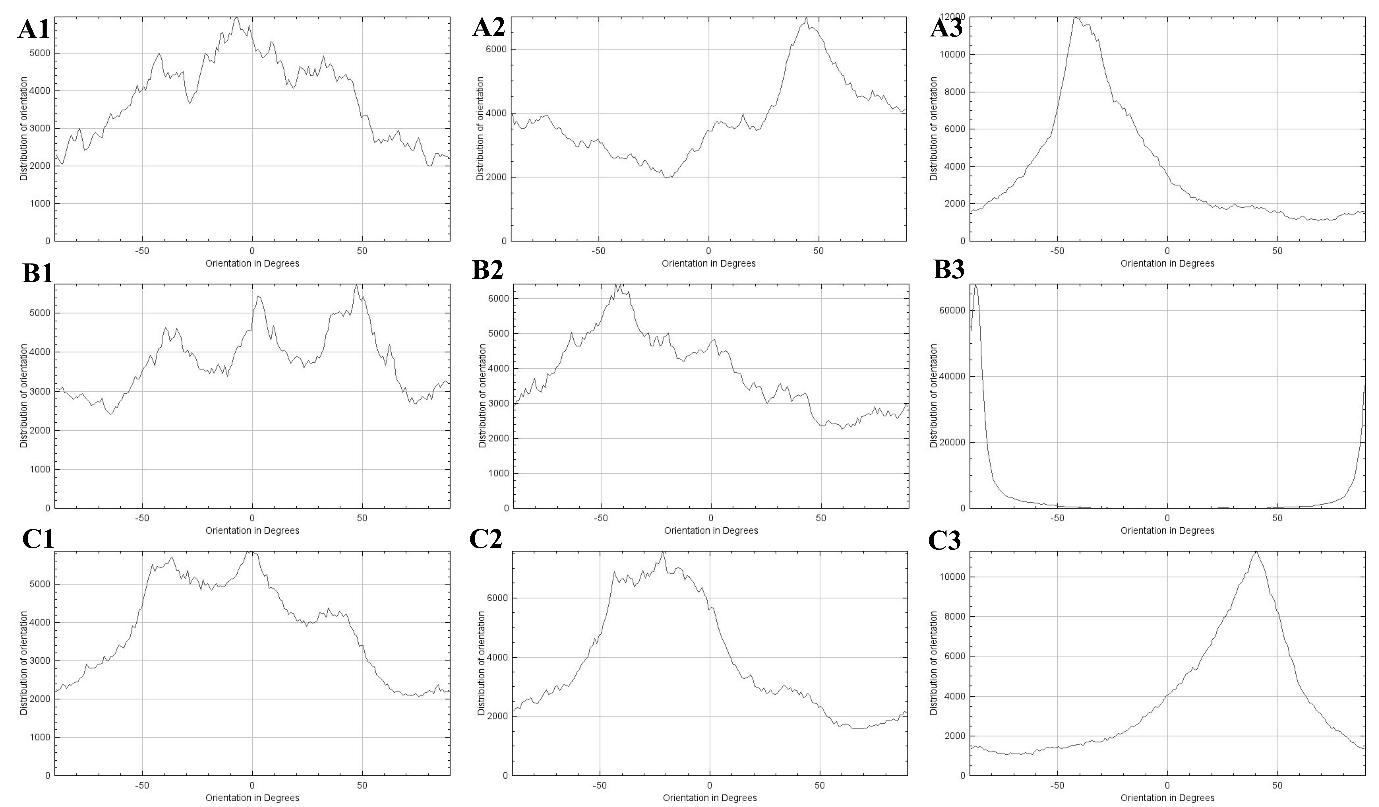


**Figure S3** Fibre orientation distribution curves (*x*-distribution of orientation and *y*-orientation in degree) for L929 cultured on PHBV film, random electrospun fibre (25 and 50 rpm), aligned fibre (150 rpm), and control (glass slide). images were analysed with ImageJ plug in OrientationJ. Phalloidin strained images were disassembled from confocal images to isolate only actin (representing cell cytoplasm) expansion on the scaffold materials. Multiple peaks of orientation degree represent less alignment with fibre orientation, whilst sharper, individual peaks represent alignment with fibre orientation (scale bar = 25 µm).


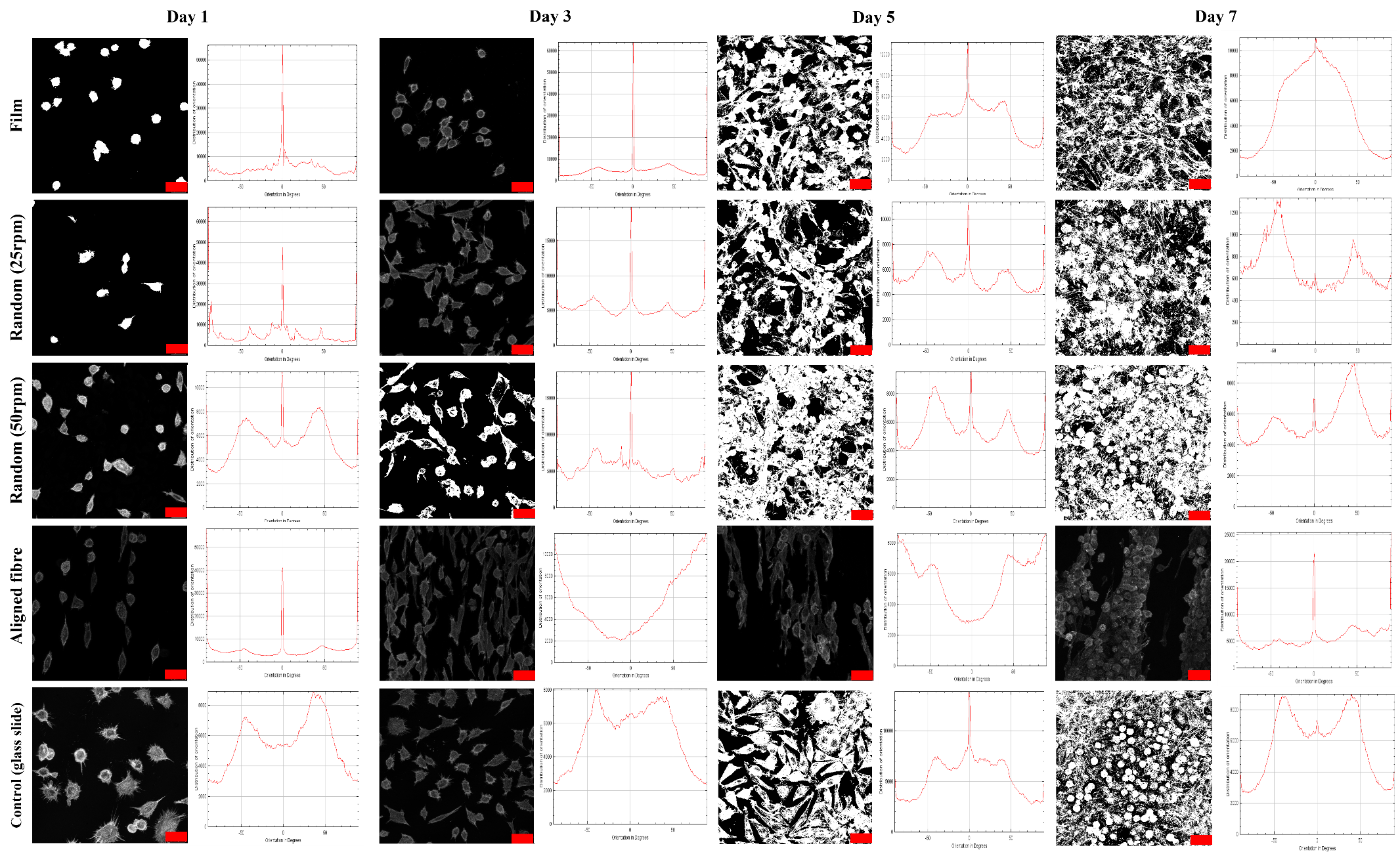

Supplement: Supplemental Material [file TSTA_A_2149034_SM6774.docx]
